# Knowledge, attitudes and practices regarding antibiotic use in Maputo City, Mozambique

**DOI:** 10.1371/journal.pone.0221452

**Published:** 2019-08-22

**Authors:** Inocêncio Mate, Charlotte Elizabeth Come, Maria Patrícia Gonçalves, Julie Cliff, Eduardo Samo Gudo

**Affiliations:** 1 National Institute of Health, Ministry of Health, Maputo, Mozambique; 2 Community Health Department, Faculty of Medicine, Eduardo Mondlane University, Maputo, Mozambique; Golden Community, NEPAL

## Abstract

**Background:**

Irrational use of antibiotics is a major driver of antimicrobial resistance (AMR) worldwide. Sub-Saharan Africa, where the risk of spread of AMR is highest, lacks data on the knowledge, attitudes and practices regarding antibiotic prescription and use. This is the first study in Mozambique to address this gap.

**Methods:**

A cross-sectional study was conducted in 2016 in 1091 adults (age ≥18 years) living in five districts in peri-urban areas of Maputo City. Three stage cluster sampling was used to select the households. A semi-structured questionnaire was used to collect information on the knowledge, attitudes and practices regarding antibiotics and their use and socio-demographic data.

**Results:**

Of the 1091 participants, 20.9% (228/1091) had used non-prescribed antibiotics. Most of the non-prescribed antibiotics were purchased in pharmacies (199/228; 87.3%). The proportion of use of non-prescribed antibiotics was higher in those who purchased from informal markets (82.6%; 14/17) and home stores (66.7%; 12/18), compared to pharmacies (24.6%; 199/810) (p = 0.000). Variables significantly associated with use of non-prescribed antibiotics were male gender (p = 0.004), living in the Central A (p<0.001), Aeroporto B (p<0.001) or 25 de Junho (p<0.001) neighborhoods, purchase of antibiotics in informal markets (p<0.002) or obtaining from home stores (p = 0.026), not completing the course (p<0.001) and having poor knowledge on the use of antibiotics (p<0.001). Main reasons for use of non-prescribed antibiotics were a perception that there was no need to attend a health facility (26.8%), followed by someone else’s advice (7.7%), symptoms similar to a previous episode (6.2%) and poor quality of care in health facilities (6.7%).

**Conclusions:**

Our study shows for the first time that knowledge regarding antibiotics is poor in Maputo City. Purchase of non-prescribed antibiotics is a common practice and most are sold in pharmacies, indicating deficient inspection. Interventions to reinforce adherence by pharmacies to current legislation for dispensing antibiotics, combined with community education are urgently needed.

## Introduction

Antibiotic resistance (AMR) is expanding at an alarming pace worldwide and represents a serious public health threat [1, 2]. Due to this rapid growth, the available options for treatment of some bacterial pathogens are almost exhausted [2, 3].

AMR is responsible for an estimated 700,000 deaths annually. By 2050, deaths may increase to 10 million deaths per year, making AMR the leading cause of death[3].

The irrational use of antibiotics, including self-medication, sub-optimal dosage, overuse, and prescription/use of inappropriate antibiotics, is considered as a major driver of the emergence and spread of AMR worldwide [4–6]. A rapid and unprecedented increase in the consumption of antibiotics has been reported globally over the last decade [7]. This increase has been greatest in low income countries, where antibiotics are more frequently dispensed without prescription [5, 8–10].

In developing countries, which bear more than 90% of the global infectious diseases burden, inappropriate use of antibiotics, including their use without prescription, is of great concern [3, 9–11]. In particular in sub-Saharan Africa, where the number of deaths attributed to AMR each year is higher than in other parts of the world [3], irrational use of antibiotics is more common [10]. In this region, legislation on antibiotic use and dispensing is often absent, and when laws do exist, they may be poorly enforced. Access to health care is often difficult and public awareness on the risk of overuse of antibiotics is limited. Within health facilities, diagnostic capacity to inform treatment is poor [8, 9, 12].

In Mozambique, current legislation prohibits the use of non-prescribed antibiotics[13]. While in the public sector, prescription of antibiotics is guided by a National Drug Formulary [14], in the private sector prescription of antibiotics often does not follow the national guidelines. However, no study has been conducted so far to assess the use of non-prescribed antibiotics as well as its associated factors.

No routine surveillance system exists to assess the profile and trends of AMR in Mozambique. However, recent publications from small studies show that AMR is increasing [8, 15, 16], with a high prevalence of multi-drug resistance [17].

A National Action Plan to Combat AMR is currently under development. Pillars of this plan include increased public awareness regarding antimicrobial resistance and rational use and prescription of antibiotics, through effective communication, education and training [2]. Evidence on the current knowledge and practices regarding use and dispensing of antibiotics is urgently needed to guide the design of these interventions. Studies in other continents have shown heterogeneous results, with few studies in sub-Saharan Africa. Lack of evidence in Mozambique stimulated this study, which aimed to investigate for the first time the knowledge, attitudes and practices towards antibiotic use among adults living in peri-urban areas of Maputo City. The study will also provide baseline data for comparison with post-intervention studies.

## Methods

### Study design and setting

A cross sectional and semi-quantitative study was conducted in peri-urban areas of Maputo City between August and November 2016. The peri-urban areas consist of large slums surrounding the urban area of the city. Most roads are unpaved, sanitation is poor, and most houses are built from rudimentary materials.

### Eligibility criteria

Eligibility criteria were: individuals aged 18 years and over, with permanent residence (> 1 year) in the selected household, who accepted to participate in the study. To mitigate recall bias, only individuals with a history of use of antibiotics for their own health or for their children´s health in the 90 days prior to the study were eligible.

### Ethics statement

This study was approved by the Institutional Bioethics Committee of the Faculty of Medicine of Eduardo Mondlane University (Ref #: CIBS FM&HCM/89/2016). Written and informed consent was obtained from each eligible individual prior to enrolment.

### Household sampling

For selection of households, we used three stage cluster sampling. In the first stage, we selected all districts of Maputo City, except Ka Tembe and Ka Nhaca, which were excluded for logistic reasons. From each district, we randomly selected neighborhoods as the second stage. In the third stage, we randomly selected at each neighborhood an imaginary center to identify the first household. From this household, consecutive households were selected one every three in a clockwise direction until the number of households for the corresponding neighborhood was reached.

In each household, only one eligible individual was interviewed according the criteria of eligibility for the study. In households with more than one eligible individual, the interviewer selected the main caretaker of children in the household.

### Sample size calculation and sampling

For sample size calculation, we used the probability proportionate to size of districts of Maputo City. Sample size was calculated using the following assumptions: i) population of Maputo City of 1,094,315 inhabitants, according to 2007 population census (CMM 2010), ii) expected frequency of use of non-prescribed antibiotics of 50%, iii) an error of the estimate of 5% and iv) a confidence interval of 95%. Based on these assumptions, our sample size was 1,111 people. We assumed a refusal proportion of 10%, giving a final sample size of 1,222 people.

To define the number of individuals to be recruited per district and neighbourhood we first obtained the weight of each district. The estimated number of people living in Maputo City (1 094 315) was divided by the estimated number of individuals living in each district. We calculated the number of individuals to be recruited in each district by multiplying our sample size (n = 1,222) by the corresponding proportion (See S1 Table).

Since we excluded two districts (Ka Nhaca and Ka Tembe), the number of individuals expected to be recruited in these two districts (n = 28) was allocated to the other five districts, according the proportion to be recruited in each of these five districts. (See S2 Table).

### Data collection

A semi-structured questionnaire (See S1 File and S2 File) was used to collect socio-demographic data and information on the knowledge, attitudes and practices regarding the use of antibiotics. The questionnaire was administered by trained interviewers. A pilot study of 60 individuals (5% of the sample size) to test all study tools was conducted. Information on monthly income is presented in Meticais (MT), the Mozambican currency. The exchange rate from US dollar to meticais during data collection was 60 (1 US dollar = 60 MT).

Information about the completeness of the course of the antibiotic was determined by comparing the course reported by the participant with that described in the National Drug Formulary.

### Identification of the antibiotic

The antibiotic used by the participant for his/her and/or their child’s health was identified by self-reporting the name and/or by showing other evidence, such as any remaining antibiotic, a medical prescription, or the antibiotic container. Participants who didn’t know the name of the antibiotic and were not able to show any evidence, were excluded from the study.

### Statistical analysis

Data from questionnaires were double entered by two separated data clerks into a data base developed using the Epidata software package version 3.1 (Epidata Association Denmark). The two datasets were then matched and all discrepancies were cleaned. Data analysis was performed using SPSS Software package version 20 (IBM, USA).

Several variables on knowledge, attitudes and practices were re-categorized and recoded. Factorial analysis was used to compare different groups after categorization of the variables. Variables related to the knowledge about antibiotics were re-categorized using the following classification “Good/Adequate if > 75%; Reasonable/Regular if ≤ 75% and > 50%, and Bad/Insufficient if ≤50%.

The chi-square test (Pearson) and logistic regression analysis were used to assess the association between socio-demographic characteristics and knowledge, attitudes and practices regarding antibiotics.

## Results

### Socio-demographic characteristics and association with use of non-prescribed antibiotics

Table 1 shows that 1091 individuals were enrolled in the study, of whom 73.1% (797/1091) were female. The median age was 33 years (IQR: 25–47). Most participants were aged 18–39 years (61.9%; 675/1091). More than 50% of the participants lived with a partner (officially married or in a de facto relationship) and 30.5% (333/1091) were single. Smaller proportions were widowed (7.2%; 79/1991) and divorced (3.0%; 33/1091). In terms of education, 50.9% (555/1091) had attained secondary level. More than 50% had a low monthly income level (less than 5,000 MT per month).

**Table 1:** Socio-demographic characteristics by use of prescribed and non-prescribed antibiotics.

Characteristics		Total	Use of prescribed antibiotics	Use of non-prescribed antibiotics	p-value
		n (%)	n (%)	n (%)	
**Total**		1091(100%)	863(79.1%)	228(20.9%)	
**Gender**					0.009
	Male	294(26.9%)	217(73.8%)	77(26.2%)	
	Female	797(73.1%)	646(81.1%)	151(18.9%)	
**Age, median (IQR)**		33(25–47)	33(25–46)	33(23–49)	0.340
**Age (years)**					0.236
	18–39	675(61.9%)	534(79.1%)	141(20.9%)	
	40–59	299(27.4%)	244(81.6%)	55(18.4%)	
	≥60	112(10.3%)	81(72.3%)	31(22.7%)	
	No information	5(0.5%)	4(80.0%)	1(20.0%)	
**Marital status**					0.027
	Single	333(30.5%)	244(73.3%)	89(26.7%)	
	Married (officially)	166(15.2%)	134(80.7%)	32(19.3%)	
	Married (de facto)	446(40.9%)	371(83.2%)	75(16.8%)	
	Widowed	79(7.2%)	64(81.0%)	15(19.0%)	
	Divorced	33(3.0%)	25(75.8%)	8(24.2%)	
	No information	34(3.1%)	25(73.5%)	9(26.5%)	
**Educational level**					0.475
	None	69(6.3%)	52(75.3%)	17(24.6%)	
	Primary	344(31.5%)	281(81.7%)	63(18.3%)	
	Secondary	555(50.9%)	436(78.6%)	119(21.4%)	
	University	114(10.4%)	86(75.4%)	28(24.6%)	
	No information	9(0.8%)	8(88.9%)	1(11.1%)	
**Monthly income (MT)**					0.271
	No income	287(26.3%)	217(75.6%)	70(24.4%)	
	<2500	241(22.1%)	203(84.2%)	38(15.8%)	
	[2500–5000]	224(20.5%)	179(79.9%)	45(20.1%)	
	[5001–10000]	183(16.8%)	143(78.1%)	40(21.9%)	
	>10000	117(10.7%)	91(77.8%)	26(22.2%)	
	No information	39(3.6%)	30(76.9%)	9(23.1)	
**Neighborhood**					<0.001
	Central A	81(7.4%)	41(50.6%)	40(49.4%)	
	Aeroporto B	176(16.1%)	136(77.3%)	40(22.7%)	
	Polana Caniço A	177(16.2%)	149(84.2%)	28(15.8%)	
	Mahotas	334(30.6%)	274(82.0%)	60(18.0%)	
	25 de Junho A	323(29.6%)	263(81.4%)	60(18.6%)	

Table 1 also shows that, out of 1091 participants, 20.9% (228) had purchased antibiotics without a prescription. In the bivariate analysis, socio-demographic characteristics associated with use of non-prescribed antibiotics were gender, marital status and place of residence. A higher proportion of males (26.2%) versus females (18.9%) (p = 0.009) used them. Use of non-prescribed antibiotics was also more frequent (p = 0.027) in single (26.7%) and divorced participants (24.2%) as compared to other marital status. A higher proportion of participants (49.4%) living in the Central A neighborhood had used non-prescribed antibiotics compared to residents in other neighborhoods (p<0.001)

Age, educational level and monthly income were not associated with use of non-prescribed antibiotics.

### Knowledge and practices regarding antibiotics and association with use of non-prescribed antibiotics

Table 2 shows that the antibiotics were mostly obtained from pharmacies (74.2%; 810/1091), with only 3.2% obtained from informal markets (1.6%) and home stores (1.6%). Most participants (94.8%) received instructions on how to use the antibiotics, but 28.7% (313/1091) did not complete the course of treatment. More than half (52.4%; 572/1091) had a poor knowledge about antibiotics and almost half (46.4%; 506/1091) had only a reasonable knowledge about use of antibiotics.

**Table 2:** Knowledge and practices regarding antibiotics by use of prescribed and non-prescribed antibiotics.

Characteristics		Total	Use of prescribed antibiotics	Use of non-prescribed antibiotics	p-value
		n (%)	n (%)	n (%)	
**Total**		1091(100%)	863(79.1%)	228(20.9%)	
**Purpose of the antibiotic being purchased**		** **	** **		<0.001
	Self-use	569(52.2%)	405(71.2%)	164(28.8%)	
	Child’s disease	290(26.6%)	255(87.9%)	35(12.1%)	
	Other household member’s disease	144(13.2%)	115(79.9%)	29(20.1%)	
	No information	88(8.1%)	88(100%)	0(0%)	
**Source of antibiotic**		** **			<0.001
	Pharmacy	810(74.2%)	611(75.4%)	199(24.6%)	
	Informal market/Other	17(1.6%)	3(17.6%)	14(82.6%)	
	Home store	18(1.6%)	6(33.3%)	12(66.7%)	
	No information	246(22.5%)	243(98.8%)	3(1.2%)	
**Received instructions for use of antibiotic?**					<0.001
	Yes	1034(94.8%)	852(82.4%)	182(17.6%)	
	No	45(4.1%)	3(6.7%)	42(93.3%)	
	Don’t remember	2(0.2%)	0(0.0%)	2(100%)	
	No information	10(0.9%)	8(80.0%)	2(20.0%)	
**Completed the course?**					<0.001
	Yes	735(67.4%)	663(90.2%)	72(9.8%)	
	No	313(28.7%)	164(52.4%)	149(47.6%)	
	Don’t remember	17(1.6%)	15(88.2%)	2(11.8%)	
	No information	26(2.4%)	21(80.8%)	5(19.2%)	
**Level of knowledge about antibiotics**					0.067
	Good	327(30.0%)	263(80.4%)	64(19.6%)	
	Reasonable	109(10.0%)	77(70.6%)	32(29.4%)	
	Poor	572(52.4%)	452(79.0%)	120(21.0%)	
	No information	83(7.6%)	71(85.5%)	12(14.5%)	
**Level of knowledge on the use of antibiotics**					<0.001
	Good	253(23.2%)	208(82.2%)	45(17.8%)	
	Reasonable	506(46.4%)	419(82.8%)	87(17.2%)	
	Poor	249(22.5%)	165(66.3%)	84(33.7%)	
	No information	83(7.6%)	71(85.5%)	12(14.5%)	

Bivariate analysis showed that frequency of use of non-prescribed antibiotics was higher in participants who obtained antibiotics for self-use (164/569; 28.8%) rather than for their children (35/290; 12.1%) or other household members (29/144; 20.1%). Most of the non-prescribed antibiotics were purchased in pharmacies (87.3%;199/228), but use of non-prescribed antibiotics was more frequent among those who obtained from informal market (14/17; 82.6%) and home stores (12/18; 66.7%) as compared to pharmacies (199/810; 24.6%). Use of non-prescribed antibiotics was also associated with lack of instruction on how to use antibiotics (93.3%; 42/45), not completing the course (47.6%, 149/ 313) and poor knowledge about the use of antibiotics (33.7%).

### Factors associated with use of non-prescribed antibiotics

Logistic regression analysis was performed to assess the variables independently associated with the use of non-prescribed antibiotics. Table 3 shows that the independently associated variables were as follows: male gender [p = 0.004, OR = 1.88 compared to reference group (female); living in the Central A neighborhood (p<0.001, OR = 4.87), Aeroporto B neighborhood (p<0.001, OR = 6.50) and 25 de Junho neighbourhood (p<0.001, OR = 3.43) compared to the reference neighbourhood (Polana Caniço A); purchasing antibiotics from informal markets (p<0.002, OR = 12.62) and from home stores (p = 0.026, OR = 4.54) compared to the reference group (pharmacy), not completing the course(p<0.001, OR = 11.42) compared to the reference group (completing the course) and having poor knowledge on the use of antibiotics (p<0.001, OR = 2.60) compared to the reference group (reasonable knowledge).

**Table 3:** Logistic regression analysis to assess the variables independently associated with use of non-prescribed antibiotics.

Characteristics		Total	Use of prescribed antibiotics	Use of non-prescribed antibiotics	OR	p-value
		n (%)	n (%)	n (%)		
**Total**		1091(100%)	863(79.1%)	228(20.9%)		
**Gender**						
	Male	294(26.9%)	217(73.8%)	77(26.2%)	1.88	0.004
	Female	797(73.1%)	646(81.1%)	151(18.9%)	-	
**Neighborhood**						
	Central A	81(7.4%)	41(50.6%)	40(49.4%)	4.87	<0.001
	Aeroporto B	176(16.1%)	136(77.3%)	40(22.7%)	6.50	<0.001
	Polana Caniço A	177(16.2%)	149(84.2%)	28(15.8%)	-	-
	Mahotas	334(30.6%)	274(82.0%)	60(18.0%)	0.59	0.088
	25 de Junho	323(29.6%)	263(81.4%)	60(18.6%)	3.43	<0.001
**Source of antibiotic**						
	Pharmacy	810(74.2%)	611(75.4%)	199(24.6%)	-	
	Informal market	17(1.6%)	3(17.6%)	14(82.6%)	12.62	<0.002
	Home store	18(1.6%)	6(33.3%)	12(66.7%)	4.54	0.026
**Completed the course?**						
	Yes	735(67.4%)	663(90.2%)	72(9.8%)	-	-
	No	313(28.7%)	164(52.4%)	149(47.6%)	11.42	<0.001
**Level of knowledge on the use of antibiotics**						
	Good	253(23.2%)	208(82.2%)	45(17.8%)	0.96	0.88
	Reasonable	506(46.4%)	419(82.8%)	87(17.2%)	-	-
	Poor	249(22.5%)	165(66.3%)	84(33.7%)	2.60	<0.001

### Socio-demographic characteristics associated with poor level of knowledge of antibiotics

Because level of knowledge about antibiotics in this study was poor, we were interested in understanding the factors associated with poor level of knowledge about antibiotics. Table 4 shows that frequency of poor level of knowledge about antibiotics increased with increasing age. Poor level of knowledge was also higher in divorced participants, in those with a lower level of education, and lower monthly income.

**Table 4:** Association between level of knowledge about antibiotics and socio-demographic characteristics.

Characteristics			Knowledge about antibiotics			p-value
		Total	Good	Reasonable	Poor	
**Total**		1008(100%)	327(32.4%)	109(10.8%)	572(56.7%)	
**Gender**						0.128
	Male	267(26.5%)	99(31.7%)	30(11.2%)	138(51.7%)	
	Female	741(73.5%)	228(30.8%)	79(10.7%)	434(58.6%)	
**Age (years)**						0.003
	18–39	637(63.1%)	226(35.5%)	75(11.8%)	336(52.7%)	
	40–59	279(27.7%)	79(28.3%)	32(11.5%)	168(60.2%)	
	≥60	87(8.6%)	21(24.1%)	2(2.3%)	64(73.6%)	
	No information	5(0.5%)	1(20.0%)	0(0%)	4(80.0%)	
**Marital status**						0.001
	Single	307(30.5%)	112(36.5%)	42(13.7%)	153(49.8%)	
	Married (officially)	144(14.3%)	62(43.1%)	13(9.0%)	69(47.9%)	
	Married (de facto)	433(43.0%)	121(27.9%)	43(9.9%)	269(62.1%)	
	Widowed	33(3.3%)	10(30.3%)	6(18.2%)	17(51.5%)	
	Divorced	66(6.5%)	16(24.2%)	2(3.0%)	48(72.7%)	
	No information	25(2.5%)	6(24.0%)	3(12.0%)	16(64.0%)	
**Educational level**						<0,001
	None	55(5.5%)	5(9.1%)	2(3.6%)	48(87.3%)	
	Primary	310(30.8%)	31(10.0%)	27(8.7%)	252(81.3%)	
	Secondary	526(52.2%)	209(39.7%)	69(13.1%)	248(47.1%)	
	University	109(10.8%)	79(72.5%)	11(10.1%)	19(17.4%)	
	No information	8(0.8%)	3(37.5%)	0(0%)	5(62.5%)	
**Monthly income (Meticais)**						<0,001
	No income	252(25.0%)	80(31.7%)	25(9.9%)	147(58.3%)	
	<2500	235(23.3%)	39(16.6%)	18(7.7%)	178(75.7%)	
	[2500–5000]	214(21.2%)	54(25.2%)	26(12.3%)	134(62.7%)	
	[5001–10000]	166(16.5%)	74(44.6%)	16(9.6%)	76(45.8%)	
	≥10000	104(10.3%)	70(67.3%)	15(14.4%)	19(18.3%)	
	No information	37(3.7%)	10(27.0%)	9(24.3%)	18(48.6%)	
**Neighborhood**						<0,001
	Central A	80(7.9%)	41(51.3%)	8(10.0%)	31(38.8%)	
	Aeroporto B	170(16.9%)	40(23.5%)	11(6.5%)	119(70.0%)	
	P. Caniço A	177(17.6%)	26(14.7%)	12(6.8%)	139(78.5%)	
	Mahotas	330(32.7)	91(27.6%)	37(11.2%)	202(61.2%)	
	25 de Junho	251(24.9%)	129(51.4%)	41(16.3%)	81(32.3%)	

Level of knowledge about antibiotics varied in different neighborhoods, with the worst knowledge observed in Polana Caniço.

### Socio-demographic characteristics associated with poor level of knowledge about the use of antibiotics

Table 5 shows that there was a trend towards increased frequency of poor knowledge about use of antibiotics with increasing age. A poor level of knowledge was also more frequent in two groups: the widowed and illiterate. Marital status (p = 0.019), educational level (p<0.001), monthly income (p < 0.001) and place of residence (p < 0.001) were associated with knowledge about use of antibiotics, but no specific trend was noted.

**Table 5:** Association between level of knowledge about the use of antibiotics and socio-demographic characteristics.

Characteristics			Level of knowledge about antibiotics			
		Total	Good	Reasonable	Poor	p-value
**Total**		1008(100%)	253(25.1%)	506(50.2%)	249(24.7%)	
**Gender**						0.774
	Male	267(24.5%)	65(24.3%)	139(52.1%)	63(23.6%)	
	Female	741(73.5%)	188(25.4%)	367(49.5%)	186(25.1%)	
**Age (years)**						0.002
	18–39	637(63.2%)	187(29.4)	296(46.5%)	154(24.2%)	
	40–59	279(27.7%)	47(16.8%)	161(57.7%)	71(25.4%)	
	>60	87(8.6%)	17(19.5%)	46(52.9%)	24(27.6%)	
	No information	5(0.5%)	2(40.0%)	3(60.0%)	0	
**Marital status**						0.019
	Single	307(30.5%)	91(29.6%)	139(45.3%)	77(25.1%)	
	Married (officially)	144(14.3%)	22(15.3%)	87(60.4%)	35(24.3%)	
	Married (de facto)	433(43.0%)	117(27.0%)	217(50.1%)	99(22.9%)	
	Widower	33(3.3%)	4(12.1%)	16(48.5%)	13(39.4%)	
	Divorced	66(6.5%)	14(21.2%)	36(54.5%)	16(24.2%)	
	No information	25(2.5%)	5(20.0%)	11(44.0%)	9(36.0%)	
**Educational level**						<0,001
	None	55(5.5%)	12(21.8%)	21(38.2%)	22(40.0%)	
	Primary	310(30.8%)	97(31.3%)	127(41.0%)	86(27.7%)	
	Secondary	526(52.2%)	132(25.1%)	282(53.6%)	112(21.3%)	
	University	109(10.8%)	10(9.2%)	72(66.1%)	27(24.8%)	
	No information	8(0.8%)	2(25.0%)	4(50.0%)	2(25.0%)	
**Monthly income (Meticais)**						<0,001
	No income	252(25.0%)	79(31.3%)	112(44.4%)	61(24.2%)	
	<2500	235(23.3%)	71(30.2%)	96(40.9%)	68(28.9%)	
	[2500–5000]	214(21.2%)	59(27.6%)	104(48.6%)	51(23.8%)	
	[5000–10000]	166(16.5%)	31(18.7%)	102(61.4%)	33(19.9%)	
	≥10000	104(10.3%)	8(7.7%)	70(67.3%)	26(25.0%)	
	No information	37(3.7%)	5(13.5%)	22(59.5%)	10(27.0%)	
**Neighborhood**						<0,001
	Central A	80(7.9%)	9(11.3%)	50(62.5%)	21(26.3%)	
	Aeroporto B	170(16.9%)	64(37.6%)	59(34.7%)	47(27.6%)	
	P. Caniço A	177(17.6%)	62(35.0%)	76(42.9%)	39(22.0%)	
	Mahotas	330(32.7%)	86(26.1%)	160(48.5%)	84(25.5%)	

### Socio-demographic characteristics associated with poor completeness of the antibiotic course

Completeness of the antibiotic course was used as a proxy to assess practice in terms of use of antibiotics and Table 6 shows that the frequency of failure to complete the course was higher in younger and in single participants. Frequency of failure was heterogeneous across different income levels (p = 0.000) and in different neighbourhoods (p<0.001) and no specific trend was noted.

**Table 6:** Association between completeness of antibiotic course and socio-demographic characteristics.

Characteristics			Completed the antibiotic course		
		Total	Yes	No	p-value
**Total**		1048(100%)	735(70.1%)	313 (29.9%)	
**Gender**					
	Male	280(26.7%)	196(70.0%)	84(30.0%)	0.506
	Female	768(73.3%)	539(70.2%)	229(29.8%)	
**Age (years)**					
	18–39	651(62.1%)	432(65.6%)	219(24.4%)	0.002
	40–59	283(27.0%)	214(75.6%)	69(22.4%)	
	>60	109(10.4%)	86(78.9%)	23(21.1%)	
	No information	5(0.5%)	3(60%)	2(40%)	
**Marital status**					
	Single	317(30.2%)	193(60.9%)	124(39.1%)	<0.001
	Married (officially)	158(15.1%)	119(75.3%)	39(24.7%)	
	Married (de facto)	430(41.0%)	313(72.8%)	117(27.2%)	
	Widower	32(2.1%)	23(71.9%)	9(28.1%)	
	Divorced	78(7.4%)	63(80.8%)	15(19.2%)	
	No information	33(3.1%)	24(72.7%)	9(27.3%)	
**Educational level**					
	None	65(6.2)	50(76.9%)	15(23.1%)	0.094
	Primary	334(31.9)	246(73.7%)	88(26.3%)	
	Secondary	531(50.7%)	354(66.7)	177(33.3%)	
	University	110(10.5%)	77(70.0%)	33(30.0%)	
	No information	8(0.8%)	8(100%)	0	
**Monthly income**					
	No income	276(26.3%)	182(68.4%)	94(34.1%)	0.005
	<2500	230(21.9%)	174(75.7%)	56(24.3%)	
	[2500–5000]	215(20.5%)	147(68.4%)	68(31.6%)	
	[5000–10000]	175(16.7%)	115(65.7%)	60(34.3%)	
	≥10000	114(10.9%)	93(81.6%)	21(28.4%)	
	No information	38(3.6%)	24(63.2%)	14(26.8%)	
**Neighborhood**					
	Central A	75(7.2%)	48(64%)	27(36%)	<0.001
	Aeroporto B	168(16.0%)	118(70.2%)	50(29.8%)	
	P. Canico A	170(16.2%)	139(81.8%)	31(18.2%)	
	Mahotas	320(30.5%)	191(59.70%)	129(40.3%)	
	25 Junho	315(30.1%)	239(75.9%)	76(24.1%)	

### Reasons for use of non-prescribed antibiotics

**Fig 1:**
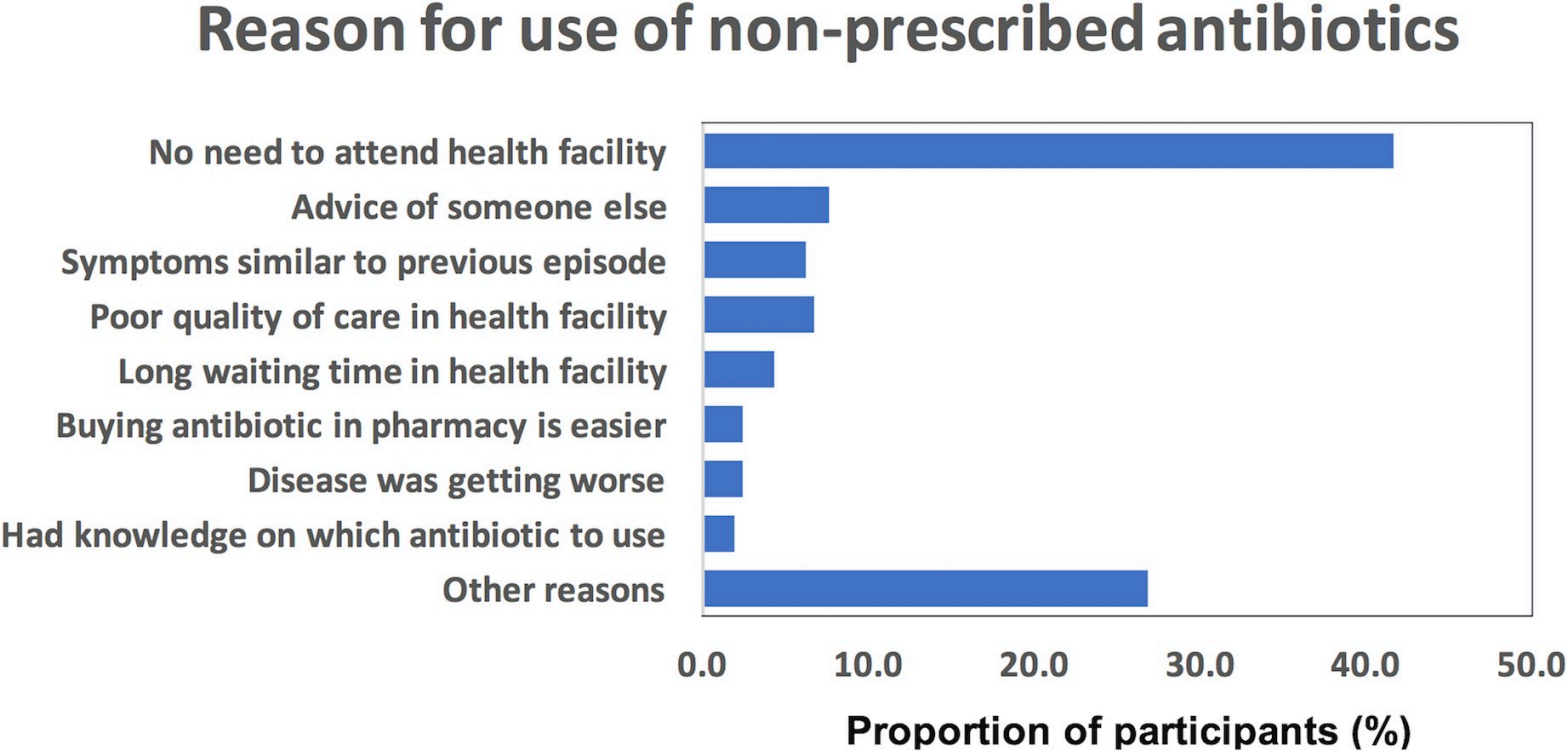
Reasons for use of non-prescribed antibiotics in those who used non-prescribed antibiotics (n = 209). Frequency represents proportion of individuals.

Fig 1 shows that the main reason for use of non-prescribed antibiotics was a perception by the participants that there was no need to attend a health facility (26.8%). Someone else’s advice (7.7%), symptoms similar to a previous episode (6.2%), poor quality of care in health facilities (6.7%) and the inconvenience of a long waiting time (4.3%) were also mentioned as reasons for use of non-prescribed antibiotics.

## Discussion

Mozambique and other countries in sub-Saharan Africa which are developing National Action Plans for combating AMR, are suffering from a paucity of data on knowledge, attitudes and practices regarding antibiotic use. In this study conducted in Maputo City, we found that use of non-prescribed antibiotics was reported by 20.9% of study participants, corroborating findings from other countries across the globe that shows that dispensing of antibiotics without prescription is a serious problem, despite being prohibited [10, 18–21]. Frequency of use of non-prescribed antibiotics found in our study are similar to those from studies conducted in Egypt and Kosovo[22, 23], but lower than those from other studies in sub-Saharan Africa [10, 20, 24].

Men used non-prescribed antibiotics more frequently than women, a finding also reported from a study in Beirut [25]. The more frequent use of prescribed antibiotics by women may be due to more contact with the formal health services because of reproductive and child health needs.

We found that adults rely more on the clinician’s recommendation for use of antibiotics for their child than when using for their own health because our data showed that use non-prescribed antibiotics was higher when the purpose was for their own health, rather than for their child´s disease.

Most non-prescribed antibiotics were obtained from pharmacies. This practice is of concern and illegal. Mozambican law prohibits dispensing of antibiotics without prescription [13]. Similar findings have been reported in other countries in sub- Saharan Africa [19, 24]. This is different from developed countries, where most non-prescribed antibiotics are obtained from family, friends and home stores [10]. This practice indicates insufficient enforcement of current legislation and poor inspection of pharmacies, which is also seen in other countries where a significant number of pharmacists were unaware of local laws and legislation on prescription of antibiotics [12, 26].

As most of non-prescribed antibiotics were bought in pharmacies, public health interventions should target not only communities, but also pharmacies and pharmacists, as recommended by others [12, 24, 27]. These interventions should focus on education of pharmacists, combined with measures to enforce existing laws, such as frequent supervisions and inspections of all pharmacies [12]. Studies in Brazil, Mexico, Thailand and Vietnam have shown that implementation of measures for enforcement of existing laws led to a reduction in over consumption of antibiotics [28, 29].

Although the proportion of antibiotics obtained from informal markets and home stores was small, the use of non-prescribed antibiotics was more frequent in individuals who obtained antibiotics from these places. Efforts should therefore be made to also inspect informal markets [12].

Use of non-prescribed antibiotics is known to be related to its incorrect use [30, 31] and data from this study showed an association between use of non-prescribed antibiotics and poor adherence to the full course of antibiotics. A similar finding was reported in Nigeria [24].

In terms of knowledge about antibiotics or their use, our data showed that more than half of the participants had a poor knowledge about antibiotics and frequency of use of non-prescribed antibiotics was significantly higher among those with poor knowledge on the use of antibiotics. Moreover, knowledge about antibiotics improved with increased educational level and monthly income. Similar results have been found in other countries [32]. Monthly income and educational level are a proxy of socio-economic status and people with higher income and education levels are likely to have better access not only to health care, but also to information [33, 34].

Although our data showed that majority of participants received instructions on the use of antibiotics, we strongly believe that instructions given to the participants were poor or inappropriate. We base this belief on the following reasons: i) a third of the participants did not complete the course, ii) more than half of the participants showed poor knowledge about antibiotics, iii) almost a quarter of the participants had poor knowledge about antibiotic use. These findings highlight the urgent need to regularly train and refresh pharmacists, along with abiding by the law and stopping the dispensing of non-prescription antibiotics.

The main reason for use/purchase of non-prescribed antibiotics was the perception that there was no need to go to a health facility. Prior experiences of poor quality of care and the inconvenience of long waiting times at health facilities were also mentioned by participants, although with less frequency. This is in agreement with findings from developing countries [35, 36], but contrasts with the reasons for self -use of antibiotics in developed countries, which are mostly related to someone else’s advice and previous experience [32, 37].

Altogether, data from this suggest that additional studies should be conducted in the country in order to assess the effectiveness and impact of integrated interventions involving different approaches, as suggested by other authors [2, 38, 39]. Previous studies in other countries have demonstrated the importance of implementation of educational programs for pharmacists and at community level and measures to increase adherence to the existing laws and legislation on the use and dispensing of antibiotics [12, 24].

This study was based only on self-reporting information by participants, which represents a limitation due to recall bias.

## Conclusions

This study expands the limited data regarding knowledge, attitudes and practices of communities regarding antibiotic use in sub-Saharan Africa and has shown for the first time that more than half of the participants had poor knowledge about antibiotics and 20.9% of participants reported they purchased non-prescribed antibiotics. This study also found that most non-prescribed antibiotics were purchased in pharmacies, despite this being legally prohibited. These findings provide evidence for the ongoing efforts in Mozambique to design interventions to reduce the use of non-prescribed antibiotics. We recommend multifaceted interventions, including reinforcement of current legislation and frequent inspections, combined with education of pharmacists and communities.

## Supporting information

S1 Table Calculation of the number of people to be enrolled from each Municipal District of Maputo City. (DOCX)

S2 Table Calculation of the number of people to be enrolled from each Municipal District of Maputo City after removal of Ka Tembe e Ka Nhaka Municipal Districts. (DOCX)

S1 File Study questionnaire in Portuguese. (DOCX)

S2 File Study questionnaire in English. (DOCX)
