# Supplementary material for: Knowledge, attitudes and practices regarding antibiotic use in Maputo City, Mozambique
Source: PLoS One. 2019 Aug 22;14(8):e0221452. doi: 10.1371/journal.pone.0221452 (PMC6705831; doi:10.1371/journal.pone.0221452)
Supplement: S1 Table — (DOCX) [file pone.0221452.s001.docx]

**S1 Table.** Table of the calculation of the number of people to be enrolled from each Municipal District of Maputo City.

| **Municipal Districts** | **# of people living in each Municipal District** | **Proportion of the corresponding population per Municipal District** | **# of people to be enrolled per Municipal District out of the 1,222 people** |
| --- | --- | --- | --- |
| DM Ka Mpfumu | 107,530 | **9.8** | **120** |
| DM Ka Lhamankulu | 155,385 | **14.2** | **174** |
| DM Ka Maxakeni | 222,756 | **20.3** | **248** |
| DM Ka Mavota | 293,361 | **26.8** | **327** |
| DM Ka Mubukuana | 290,696 | **26.6** | **325** |
| DM Ka Tembe | 19,371 | **1.8** | 22 |
| DM Ka Nhaka | 5,216 | **0.5** | 6 |
| **Maputo city** | **1,094,315** | **100** | **1,222** |
