# Supplementary material for: Knowledge, attitudes and practices regarding antibiotic use in Maputo City, Mozambique
Source: PLoS One. 2019 Aug 22;14(8):e0221452. doi: 10.1371/journal.pone.0221452 (PMC6705831; doi:10.1371/journal.pone.0221452)
Supplement: S2 Table — (DOCX) [file pone.0221452.s002.docx]

**S2 Table.** Table of the calculation of the number of people to be enrolled from each Municipal District of Maputo City after removal of Ka Tembe e Ka Nhaka Municipal Districts.

| **Municipal Districts** | **# of people to be enrolled from each Municipal District** | **Redistribution of people from Ka Tembe and Ka Nhaca Districts** | **# of people to be enrolled per Municipal District after removal of Ka Tembe and Ka Nhaca Districts** |
| --- | --- | --- | --- |
| DM Ka Mpfumu | 120 | 3 | **123** |
| DM Ka Lhamankulu | 174 | 4 | **178** |
| DM Ka Maxakeni | 248 | 6 | **254** |
| DM Ka Mavota | 327 | 8 | **335** |
| DM Ka Mubukuana | 325 | 7 | **332** |
| DM Ka Tembe | 22 | - | **-** |
| DM Ka Nhaka | 6 | - | **-** |
| **Maputo City** | - | - | **-** |
