# Supplementary material for: Knowledge, attitudes and practices regarding antibiotic use in Maputo City, Mozambique
Source: PLoS One. 2019 Aug 22;14(8):e0221452. doi: 10.1371/journal.pone.0221452 (PMC6705831; doi:10.1371/journal.pone.0221452)
Supplement: S1 File — (DOCX) [file pone.0221452.s003.docx]

FICHA DE ENTREVISTA **PARA PARTICIPANTES DAS COMUNIDADES**

ESTUDO: Avaliação de conhecimentos e práticas de prescrição de antibióticos pelos profissionais de saúde, e de uso dos mesmos pelas comunidades, na Cidade de Maputo, 2016.

Avaliação das práticas de utilização de antibióticos pelos profissionais de saúde e pelas comunidades na cidade de Maputo, 2015.

Bairro : _____________ _________

**I. IDENTIFICAÇÃO DOS INQUIRIDOS**

1. Data __/__/20___;

2. Código __ __ __ __

3. Idade __ __ ; 4. Sexo: F __ M__

5. Profissão ___________________________

6. Nível de escolaridade:

Primário __ Secundário ___ Pré-Universitário ___ Superior __

7.Estado Civil: Solteiro (a) __ Casado (a) ___ Divorciado (a) ___ Viúvo (a) ___

**II. INFORMAÇÃO SOBRE UTILIZAÇÃO DE MEDICAMENTOS (ANTIBIÓTICOS)**

1. Usou algum medicamento nos últimos 30 dias? Sim ___ Não ____

2. Se sim, qual/quais os medicamentos que utilizou?_____________________________________

3. Usou alguma receita para aquisição desse medicamento? Sim ___ Não ___

4. Adquiriu algum medicamento sem receita? Sim ___ Não ____

5. Se sim, qual ou quais?_______________________________________________________

6. Onde obteve os medicamentos?

a) Farmácia _____ b) Outro___________________________________

7. Como identificou esses medicamentos?

a) já os conhecia ___ b) por indicação de alguém ___ c) Usei receita antiga ___

d) Outro______________________________

8. Qual o motivo (doença) que o levou a usar o medicamento? ___________________________________________________________________________

**A. IDENTIFICAÇÃO DOS INQUIRIDOS**

1. Data: |__|__|20___|

2. Código |___| |__| |__| |__| 3. Bairro|_________________________|

4. Idade: |__|__| 5. Sexo: F |__| M|__|

6. Ocupação |_____________________|

7. Nível educacional: Sem escolaridade |__| Primário |__| Secundário |__| Pré-Universitário |__| Superior |__|

8. Estado Civil: Solteiro (a) |__| Casado (a) |__| Vive maritalmente |__|

Divorciado (a) |__| Viúvo (a) |__|

9. Rendimento médio mensal

Sem rendimento |__| Inferior a 2500,00 Mt |__| 2500,00 Mt a 5000,00 Mt |__| 5000,00 Mt a 10 000,00 Mt |__| 10 000,00Mt a 20 000,00MT|__| 20 000,00 Mt a 30 000,00Mt |__| Mais de 30 000,00 Mt|__|

**B. INFORMAÇÃO SOBRE USO DE MEDICAMENTOS (ANTIBIÓTICOS)**

**I. Práticas de uso de antibióticos**

1. Usou algum medicamento (antibiótico) nos últimos 90 dias? a) Sim |__| b) Não |__|

2. Se sim, qual/quais os antibióticos utilizou?|____________________________________

__________________________________________________________________________|

3. O antibiótico utilizado foi obtido por prescrição médica?

a) Sim |__| b) Não |__|

4. Caso tenha adquirido sem receita médica, como identificou esses antibióticos?

a) já os conhecia |__| b) por indicação de alguém |__| c) Usei receita antiga |__|

d) Outro (qual?) |______________________________|

5. Qual o motivo que o (a) levou usar o antibiótico sem receita médica?|________________

__________________________________________________________________________|

6. Para quem foi usado o antibiótico?

a) o próprio |__| b) Meu filho |__| c) Outro |__|

7. Quem decidiu se deveria tomar o antibiótico?

a) o próprio |__| b) um amigo/colega |__| c) um médico ou PS |__| d) um familiar |__| e) um farmacêutico |__| f) Outro |__|

8. Onde adquiriu os antibióticos?

a) Farmácia |__| b) Supermercado |__| c) Mercado negro |__|

d) Já existia em casa |__| e) Alguém forneceu |__| f) Outro |__|

9. Recebeu instrução de como tomar os antibióticos?

a) Sim |__| b) Não |__| c) Não lembro |__|

10, Tomou a dose completa do(s) antibiótico(s)?

a) Sim |__| b) Não |__| c) Não lembro |__|

10.1 Se sim, qual foi? |___|___|

11. Para que tratamento usou o antibiótico?|______________________________________|

**I. Conhecimentos sobre uso de antibióticos**

1. Antibiótico e anti-inflamatório são a mesma coisa?

a) Sim |__| b) Não |__| c) Não sei |__|

2. O que são antibióticos? |_____________________________________________________

__________________________________________________________________________|

3. Quais as infecções que os antibióticos servem para curar?

a) Bactérias |__| b) Vírus|__| c) outros(quais?)|__| d) Não sei |__|

4. Qual é a sua opinião em relação em relação a aquisição de antibióticos sem prescrição médica?

a) Podem ser adquiridos |__| b) Não devem ser adquiridos |__| c) Não tenho opinião |__|

Outra |__|

5. Qual é a sua opinião em relação ao consumo excessivo de antibióticos? que o consumo excessivo de antibióticos pode ser prejudicial a saúde?

a) É prejudicial |__| b) Não é prejudicial |__| c) Não tenho opinião |__| Outra |__|

6. Quais os riscos que acha que podem ocorrer pelo consumo de antibióticos sem orientação médica? |___________________________________________________________________|

a) Nenhum |__| b) Falha no tratamento |__| c) Complicação da saúde do paciente|__| d) Surgimento de outras doenças|__| e) Não sei|__| f) Outro (qual?) |________________|
