# Supplementary material for: Knowledge, attitudes and practices regarding antibiotic use in Maputo City, Mozambique
Source: PLoS One. 2019 Aug 22;14(8):e0221452. doi: 10.1371/journal.pone.0221452 (PMC6705831; doi:10.1371/journal.pone.0221452)
Supplement: S2 File — (DOCX) [file pone.0221452.s004.docx]

PARTICIPANT’S INTERVIEW QUESTIONAIRE

Study title: Knowledge, attitudes and practices regarding antibiotic use in Maputo City, Mozambique

Bairro : _____________ _________

**I. IDENTIFICAÇÃO DOS INQUIRIDOS**

1. Data __/__/20___;

2. Código __ __ __ __

3. Idade __ __ ; 4. Sexo: F __ M__

5. Profissão ___________________________

6. Nível de escolaridade:

Primário __ Secundário ___ Pré-Universitário ___ Superior __

7.Estado Civil: Solteiro (a) __ Casado (a) ___ Divorciado (a) ___ Viúvo (a) ___

**II. INFORMAÇÃO SOBRE UTILIZAÇÃO DE MEDICAMENTOS (ANTIBIÓTICOS)**

1. Usou algum medicamento nos últimos 30 dias? Sim ___ Não ____

2. Se sim, qual/quais os medicamentos que utilizou?_____________________________________

3. Usou alguma receita para aquisição desse medicamento? Sim ___ Não ___

4. Adquiriu algum medicamento sem receita? Sim ___ Não ____

5. Se sim, qual ou quais?_______________________________________________________

6. Onde obteve os medicamentos?

a) Farmácia _____ b) Outro___________________________________

7. Como identificou esses medicamentos?

a) já os conhecia ___ b) por indicação de alguém ___ c) Usei receita antiga ___

d) Outro______________________________

8. Qual o motivo (doença) que o levou a usar o medicamento? ___________________________________________________________________________

**A. PARTICIPANTS IDENTIFICATION**

1. Date: |__|__|20___|

2. Code |___| |__| |__| |__| 3. Neighborhood|_________________________|

4. Age: |__|__| 5. Gender: Female |__| Male|__|

6. Ocupation |_____________________|

7. Educational level: No Education |__| Primary |__| Secondary |__| Pre-University |__| University |__|

8. Marital status: Single |__| Married) |__| Live martially |__|

Divorced __| Widowed |__|

9. Monthly income

Sem rendimento |__| < 2500,00 Mt |__| 2500,00 Mt to 5000,00 Mt |__| 5000,00 Mt to 10 000,00 Mt |__| 10 000,00Mt to 20 000,00MT|__| 20 000,00 Mt to 30 000,00Mt |__| > 30 000,00 Mt|__

**B. INFORMATION ABOUT ANTIBIOTIC USE**

**I. Practices of antibiotics use**

1. Did you use any antibiotics in the last 90 days?

a) Yes |__| b) No |__|

2. If yes, which antibiotics did you use?|____________________________________

__________________________________________________________________________|

3. The antibiotic used was obtained with medical prescription?

a) Yes |__| b) No |__|

4. If the antibiotics were obtained with non-prescription, how did you get them?

a) I know about this antibiotics |__| b) Someone advised |__| c) I used the old prescription |__| d) Other (which one?) |______________________________|

5. What was the reason for using antibiotics with non-prescription?|________________

__________________________________________________________________________|

6. To whom was the antibiotics used?

a) Myself |__| b) My child |__| c) Other |__|

7. Who decided you should take the antibiotic?

a) Myself |__| b) a friend |__| c) a Medical doctor or health professional |__| d) a parent |__| e) a pharmacist|__| f) Other |__|

8. Where did you get or purchased?

a) Pharmacy |__| b) Supermarket |__| c) Informal market |__|

d) In home store |__| e) Someone provided |__| f) Other|__|

9. Have you received the instructions on how to take the antibiotics?

a) Yes |__| b) No |__| c) Don’t remember |__|

10, Did you take full dose of antibiotic or antibiotics?

a) Yes |__| b) No |__| c) Don’t remember |__|

10.1 If yes, which one? |___|___|

11. For which treatment did you use the antibiotics?|_________________________________|

**I. Knowledge of antibiotics use**

1. Is antibiotic and anti-inflammatory the same thing?

a) Yes |__| b) No |__| c) Don’t know|__|

2. What is antibiotic? |_____________________________________________________

__________________________________________________________________________|

3. Which diseases antibiotics can be treated by antibiotics?

a) Bacterial |__| b) Virus|__| c) others (which one?)|__| d) Don’t know |__|

4. What is your opinion regarding obtaining antibiotics with non-prescription?

a) May be obtained |__| b) May not be obtained |__| c) I’ve no opinion |__|

Other |__|

5. What is your opinion regarding the excessive consumption of antibiotics?

a) It’s bad for health |__| b) It’s not bad |__| c) I’ve no opinion |__| Other |__|

6. Which risks can occur when consuming antibiotics without nonprescription?

a) None |__| b) Failure of treatment |__| c) Patient’s health complications|__| d) Emerging of new diseases|__| e) Don’t know__| f) Other (which one?) |________________|
